# Suspected Pulmonary Metastasis of Actinic Cutaneous Squamous Cell Carcinoma

**DOI:** 10.1155/2017/4176071

**Published:** 2017-03-13

**Authors:** Monet E. Meter, David J. Nye, Christian R. Galvez

**Affiliations:** ^1^Department of Medicine, Sky Ridge Medical Center, Lone Tree, CO, USA; ^2^Department of Surgery, Berkshire Medical Center, Pittsfield, MA, USA

## Abstract

*Introduction.* It is rare for actinic or squamous cell carcinoma (SCC) in situ to metastasize.* Case Presentation.* A 67-year-old male had a significant medical history including severe psoriatic arthritis treated with UVB, methotrexate, and rapamycin. He had twenty-five different skin excisions of actinic keratosis four of which were invasive SCC. Our patient developed shortness of breath necessitating a visit to the emergency department. A CT scan of his chest revealed a mass in the right lower lung. A subsequent biopsy of the mass revealed well-differentiated SCC. He underwent thoracoscopic surgery with wedge resection of the lung lesion.* Discussion.* Actinic keratosis (AK) is considered precancerous and associated with UV exposure. It exists as a continuum of progression with low potential for malignancy. The majority of invasive SCCs are associated with malignant progression of AK, but only 5–10% of AKs will progress to malignant potential.* Conclusion.* In this case, a new finding of lung SCC in the setting of multiple invasive actinic cutaneous SCC associated with a history of extensive UV light exposure and immunosuppression supports a metastatic explanation for lung cancer.

## 1. Introduction

Cutaneous squamous cell carcinoma (SCC) is the second most common skin cancer [1], accounting for 20% of all cutaneous malignancies [2], with risk factors that include accumulation of sun and other UV exposure, and immunocompromised [2]. It is the most common cancer capable of metastatic spread [3]. SCC includes many histopathologic subtypes, each associated with various behaviors, courses, and prognoses [1, 2, 4]. Although potential to metastasize is increased for high grade subtypes such as nonactinic SCC, it rarely occurs in patients with actinic or SCC in situ [1, 2], with only 5–10% ever progressing to invasive SCC over a five-year period [2, 4]. In this case, a new finding of lung SCC in the setting of multiple invasive actinic cutaneous SCC and history of extensive UV light exposure and immunosuppression supports a metastatic explanation for the lung cancer.

## 2. Case Presentation

The patient is a 67-year-old male with severe psoriatic arthritis involving his entire body but sparing his face, who has undergone 800+ UVB treatments over his 40-year-history of the condition and who has likewise been treated at various times with methotrexate and rapamycin for this autoimmune disease. He also has a long and significant past medical history of multiple, recurrent cutaneous SCC. Twenty-five of these lesions have been removed, nine of which were within the past 2 years, and at least four of which were invasive, albeit resected with negative margins. At least one lesion was classified as actinic keratosis.

**Figure 1:**
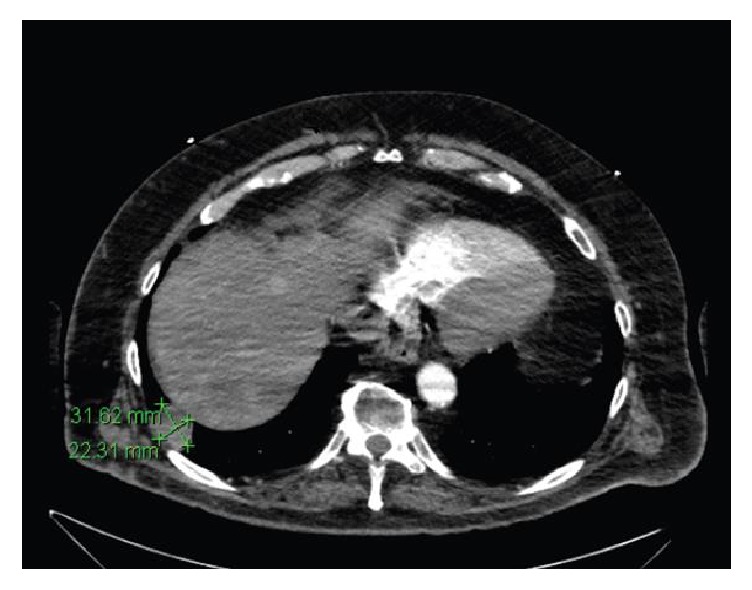
Computed tomography angiography of the thorax showing well-circumscribed, ovoid “cannonball” costophrenic mass in right lower lung lobe, consistent with appearance of metastasis.

He recently presented to the BMC Emergency Department complaining of sudden onset shortness of breath after exertion, without chest pain, wheezing, productive cough, or calf pain. Given his family history of thromboembolic disease and his own remote history of deep vein thrombosis with subsequent pulmonary embolism (PE), his care team moved to rule out a recurrence of this process and possible hypercoagulable disorder. Labs showed troponins were within normal limits, but an elevated D-dimer of 437 ng/mL. An EKG showed normal sinus rhythm with multiple PACs. A chest X-ray revealed mild cardiomegaly but no infiltrates. A computed tomography angiography (Figure 1) found no PE but revealed an ovoid 3.2 × 2.2 cm costophrenic angle mass of the right lower lung lobe with no evidence of hilar or mediastinal lymphadenopathy. His symptoms resolved without treatment. He was stable on discharge and referred to a pulmonary specialist for evaluation of the lung nodule.

**Figure 2:**
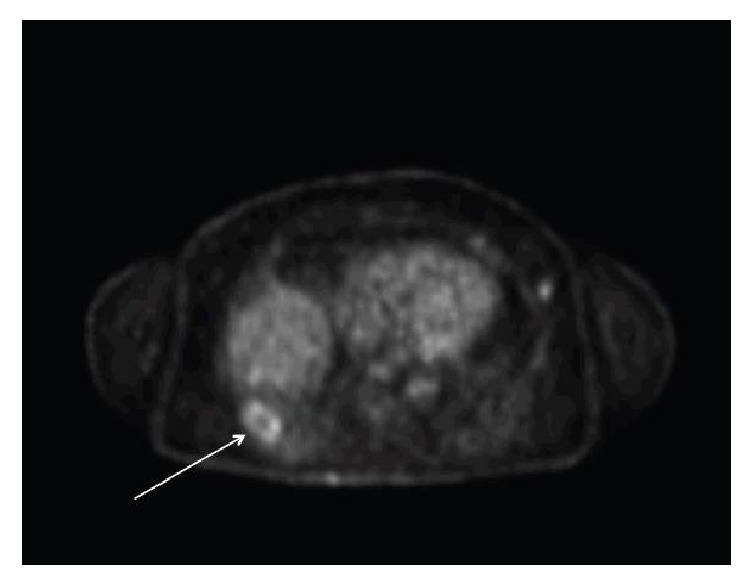
PET scan identifies costophrenic lung mass as peripherally hypermetabolic with relatively ametabolic central region, likely cavitation from necrosis (max SUV 3.9).

A month later, a CT-guided biopsy was performed by Interventional Radiology, which confirmed the diagnosis of keratinizing, well-differentiated SCC and extensive necrosis. Of note were several scattered lesions of cutaneous SCC on the patient's abdomen and back at the time of this procedure, one of which was oozing, that the patient did not believe required attention at this time. Given the question of metastasis, a staging positron emission tomography (PET) scan was performed, revealing hypermetabolic foci in addition to the known lung mass with a max standardized uptake value (SUV) of 3.9 (Figure 2). SUV is a measurement of how metabolically active a tissue is. As the SUV increases there is a higher association with cancer.

**Figure 3:**
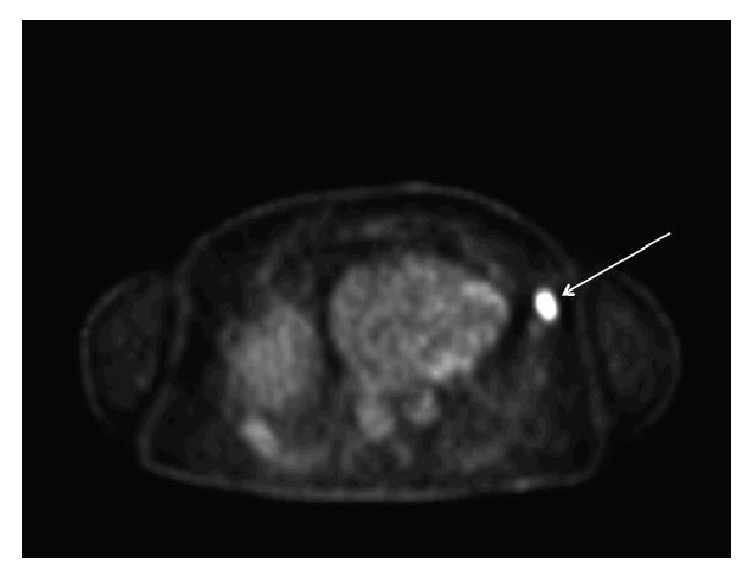
PET scan identifies intensely hypermetabolic nodular lesion on the left lateral chest wall inferior to left axilla, highly suspicious for malignancy (max SUV 12.5).

**Figure 4:**
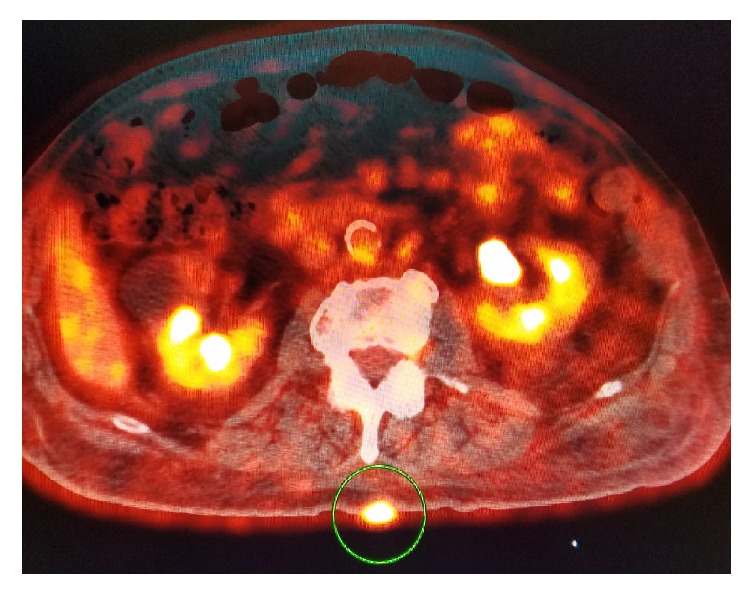
PET scan identifies intensely hypermetabolic cutaneous lesion of the back. This patient has a history of multiple hypermetabolic cutaneous lesions with excisions.

- Intensely hypermetabolic nodular lesion within the subcutaneous soft tissue of the left anterior/inferior chest wall inferior to left axilla measuring 2.6 × 1.7 cm—suspicious for malignant lymph node (Figure 3).
- At least seven additional scattered cutaneous nodular foci in the right and left superior back, right inferior back, and right and bilateral inferior midline areas of the anterior abdomen, any of which could represent SCC (Figure 4).

**Figure 5:**
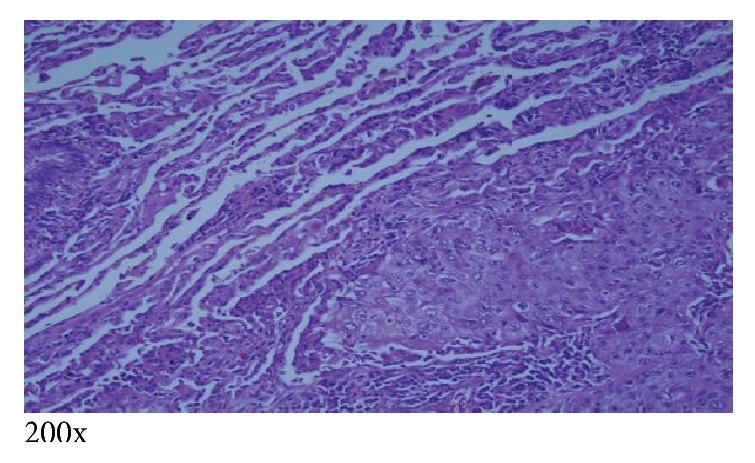
Normal lung tissue with intact alveolar spaces adjacent to the keratinizing squamous cell carcinoma, which, here, is more moderately differentiated and more clearly characteristic of SCC [2]. The tumor measures 4.5 × 3.7 × 2.2 cm, and the distal border of the tumor invades the pleura.

**Figure 6:**
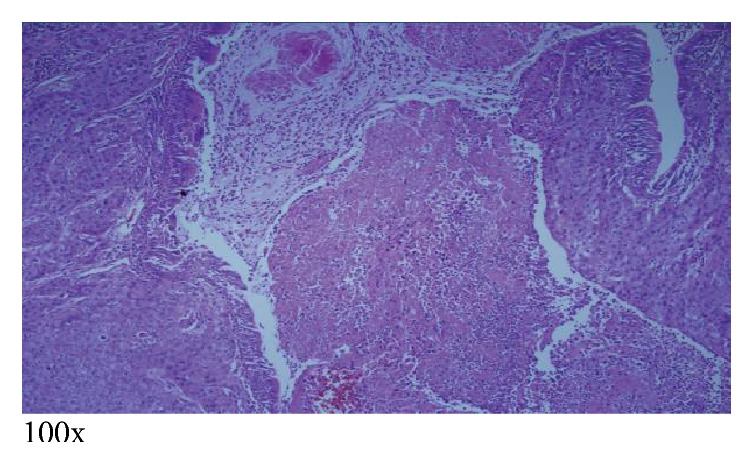
SCC invasion of bronchus. Note the intact pseudostratified ciliated columnar epithelium of the bronchial wall to the left and upper right of the image, while the lower right portion shows the tumor breaking through the bronchial wall and invading the bronchial lumen.

**Figure 7:**
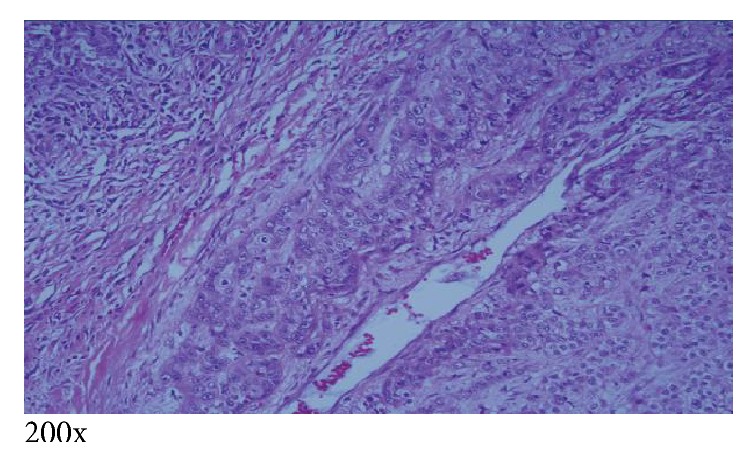
Angioinvasion—SCC has engulfed all muscles layers of a large blood vessel wall. Only the endothelium of the tunica intima remains intact.

**Figure 8:**
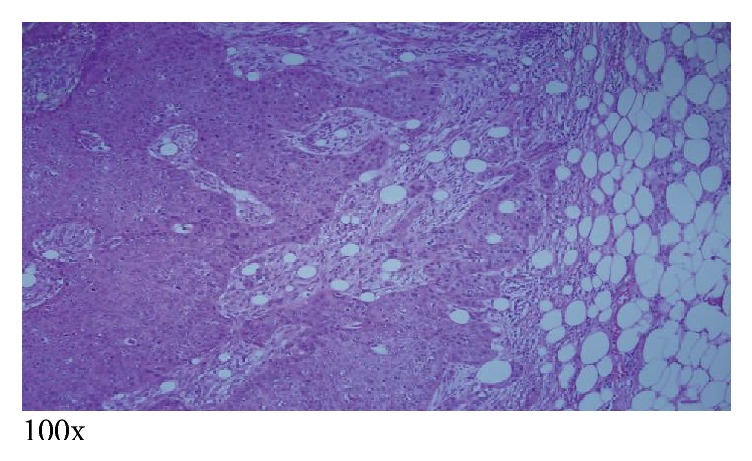
Left chest invasive cutaneous SCC measuring 9.8 cm, excised a few years ago, revealing desmoplastic reaction and deep invasion into subcutaneous fat, a strong predictor of metastasis [1]. Moderately differentiated.

**Figure 9:**
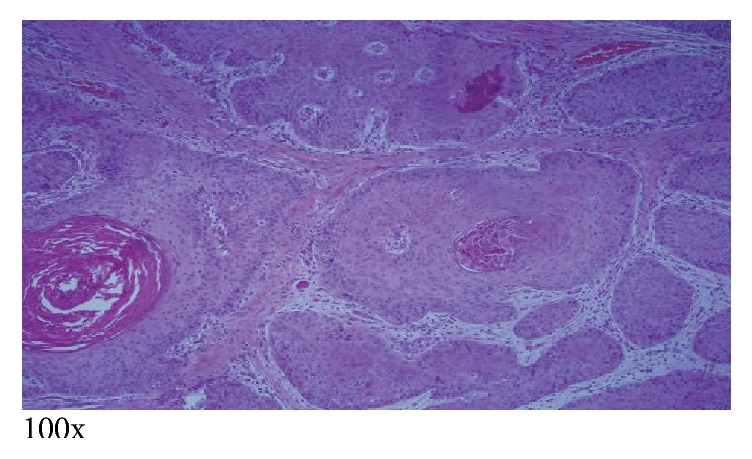
Right shoulder invasive cutaneous SCC measuring 7.8 cm, excised a few years ago, showing “nests” of deep dermal and subcutaneous invasion [2].

The patient subsequently underwent right subxiphoid video-assisted thoracoscopic surgery (VATS) in which a wedge excision of the right lower lobe was performed. A thoracostomy tube was inserted. Excision of the left chest wall nodule was also performed. The lung SCC (Figures 5–7) is histopathologically similar to biopsies of prior cutaneous SCC (Figures 8 and 9). The right lower lung mass was negative when stained with TTF-1. TTF-1 would stain positive for adenocarcinoma. A p63 stain would identify SCC from any site and that was positive on the lung sample. TTF-1 and p63 staining was not performed on the cutaneous SCC lesions. There are no markers that would identify or exclude a primary lung tumor that has metastasized to the skin.

**Figure 10:**
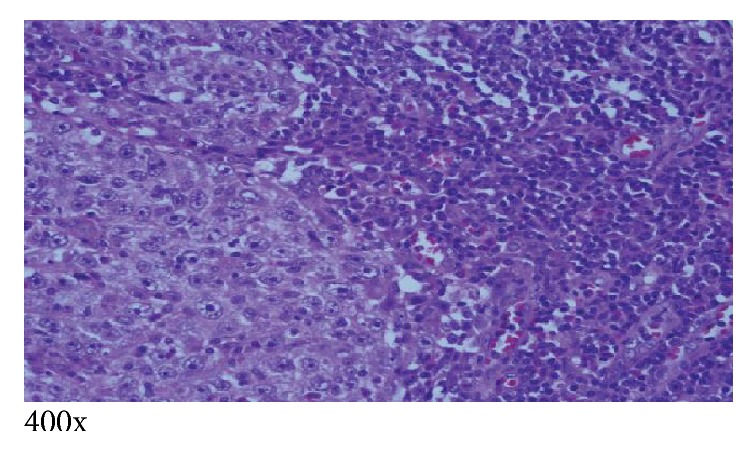
Left chest wall lymph node measuring 3.0 × 2.5 × 1.7 cm poorly differentiated, higher grade squamous cell carcinoma. Extranodal invasion is suspected but not definitive.

Prior to completion of the pathology report, it was unclear if the left anterior/inferior lymph nodule malignancy is in fact a metastasis, related to the right lower lobe lung SCC, or a separate, primary, synchronous tumor such as a skin carcinoma. Pathologic examination of the nodule did show it to be of histologically higher grade than the lung or skin SCC (Figure 10). The lung was TTF-1 negative for adenocarcinoma and p63 positive for SCC.

## 3. Discussion

The singularity of this lung lesion might suggest its occurrence as a primary SCC of the lung. However, the appearance on preoperative imaging (Figure 1) suggests metastasis from any of numerous primary cutaneous SCC due to its very well-circumscribed edges and “cannonball” appearance. A primary SCC of the lung would typically appear on CT imaging as a speculated lesion. The location of the lesion is also in the periphery of the lung, while this type of lung cancer usually arises centrally with hilar vessel and bronchiolar invasion. Additionally, primary SCC of the lung is highly associated with cigarette smoking or other environmental exposure [2], of which this patient has no history.

Actinic keratosis (AK) is considered precancerous and associated with UV exposure but exists as a continuum of progression with low potential for malignancy [2]. The majority (97%) of invasive SCCs are associated with malignant progression of AK, but only 5–10% of AKs will progress to malignant potential [2]. However, even invasive SCC is considered indolent, and metastatic potential is still only 0.5% [2]. More poorly differentiated SCC and lesions over 2 cm possess increased potential to metastasize [1, 2, 4] and, in this group, do so at a rate of 11–47.3% [1]. Dermal and subcutaneous involvement increase probability of metastasis sevenfold [2]. In this case, despite the absence of nonactinic SCC with higher metastatic potential, the risk for metastasis in this patient is exponentially increased given his unique set of risk factors: continued exposure to UV light, his immune-suppressing drug regiment, his propensity to develop multiple, recurrent primary cutaneous SCCs with invasive qualities, his history of very large (~3–9 cm width) and deep cutaneous tumors, and his self-reported delay to treatment of his present cutaneous lesions [2, 4].

The right lower lung mass was negative when stained with TTF-1. TTF-1 would stain positive for adenocarcinoma [5]. A p63 stain would identify SCC from any site and that was positive on the lung sample. TTF-1 and p63 staining was not performed on the cutaneous SCC lesions. There are no markers that would identify or exclude a primary lung tumor that has metastasized to the skin.

No markers are currently in use to reliably distinguish primary versus secondary SCC, as both cutaneous and lung SCC possess the same measured markers [3, 6] although the novel Pathfinder TG, Metastases versus Primary Tumors (MvP) test developed by RedPath Integrated Pathology, may be useful in this case.

## 4. Conclusion

Given this patient's unique set of circumstances and risk factors—the size, invasive nature and sheer number of his past skin tumors, the location and radiographic appearance of the lung mass, and histologic similarity to his cutaneous SCC—the possibility of a rare occurrence of metastasis of actinic cutaneous SCC to the lung from prior or current cutaneous SCC is plausible. Despite being a solitary lesion, the patient's negative history of smoking or environmental exposure would make a primary lung SCC less likely.
